# Embryo Transfer Procedural Parameters Do Not Predict IVF Cycle Outcome

**DOI:** 10.3390/jcm13051312

**Published:** 2024-02-26

**Authors:** Konstantinos Sfakianoudis, Evangelos Maziotis, Anna Trypidi, Sokratis Grigoriadis, Terpsithea Vaxevanoglou, Irene Angeli, Anna Rapani, Amalia Kotsifaki, Kalliopi Pistola, Agni Pantou, Konstantinos Dafopoulos, Konstantinos Pantos, Mara Simopoulou

**Affiliations:** 1 Genesis Athens Clinic, Centre for Human Reproduction, Papanikoli, 15232 Athens, Greece; 2 Department of Physiology, Medical School, National and Kapodistrian University of Athens, 11527 Athens, Greece; 3 Department of Obstetrics and Gynecology, School of Health Sciences, Faculty of Medicine, University of Thessaly, 41500 Larisa, Greece; kdafop@yahoo.com

**Keywords:** assisted reproduction, IVF, embryo transfer, outcome, pre-implantation embryo

## Abstract

Background: this study aims to assess the effect of embryo transfer (ET) performance parameters of a technical nature on IVF outcome. Methods: A total of 1417 ETs from a single IVF center were included in this prospective observational study. The parameters investigated were as follows: the presence of cervical mucus post catheter withdrawal, the presence of blood, catheter reload, the employment of a tenaculum and stylet, catheter resistance as experienced by the physician and patient discomfort. Results: When ET performance parameters were associated with clinical outcomes on a singular level, none of the ET parameters presented with any statistical significance. The evaluation of covariates indicated that the number and the quality of transferred embryos, as well as maternal age, exerted a statistically significant effect on clinical outcomes. In a multivariate analysis, only the presence of mucus along with significant catheter resistance presented with statistical significance; however, when adjusting for covariates, this combination showed no statistically significant effect on clinical outcomes. Conclusions: the results indicate that the time-consuming process of recording and analyzing ET performance parameters fails to offer any additional value in predicting the cycle’s outcome, while factors like embryo quality and number, as well as maternal age, seem to be the sole robust predictive factors of an IVF cycle.

## 1. Introduction

Embryo implantation constitutes a complex process entailing multiple biological factors [1], and embryo transfer (ΕΤ) represents a crucial step in in vitro fertilization (IVF). Numerous studies focusing on embryo competence and endometrial status have been conducted [2], and it has been established that a good-quality embryo, a receptive endometrium and an effective dialogue between them are required to achieve pregnancy [1]. The timing of ET, which can take place from day two to day seven post oocyte fertilization, is critical for the success of implantation [3], and the debate and reasoning behind each option are dynamically discussed in the literature [4,5], while molecular data have been argued to be useful in indicating the optimal ET day within the menstrual cycle, albeit a more personalized approach seems to be required [6].

ET procedural parameters of a technical nature and practices that physicians and embryologists perform during embryo transfer have been reported to affect IVF outcome [2]. Ultrasound imaging which facilitates embryo transfer through the visualization of the catheter placement within the endometrial cavity, is established as an effective method of embryo transfer [7]. According to ASRM guidelines, transabdominal ultrasound-guided embryo transfer has been recommended to enhance clinical pregnancy rate and live-birth rate [8].

Several studies investigate the impact of the presence of mucus and blood on the catheter, following its withdrawal, on clinical outcomes. It has been reported that cervical mucus may impair the success rates of IVF, blocking the transfer catheter tip and inducing embryo retention [8]. On the other hand, the majority of studies indicate that the presence of cervical mucus on the catheter is not associated with adverse effects on clinical outcomes, and this is based on fair evidence [8]. Furthermore, the presence of blood on the catheter is generally considered to be an indication of cervical or endometrial bleeding due to a traumatic transfer, which may impair implantation [9]. The literature has presented conflicting results regarding the effect of the presence of blood on the catheter [8].

When reporting on the difficulty of ET, another level of complexity is introduced. The literature presents discrepant definitions on what constitutes an easy or difficult ET [10]. The term “difficult ET” may hold different meanings with regards to different parameters of ET that have been investigated in each study [11]. This may pose a concern for the scientific community when communicating data and conclusions. In the absence of a blanket definition, it may be essential to reframe “difficulty” on specific characteristics of ET, enhancing the objectivity of the interpretation of the outcome. It has been suggested that the presence of blood may be a marker of difficulty of transfer [12]. Difficult transfers are identified according to some practitioners as those presenting with resistance regarding the progress of the transfer catheter [11]. Contrarily, an easy transfer is performed smoothly without requiring cervical manipulation and without blood on the transfer catheter [11]. Difficult transfers have been described as requiring additional time or effort, firmer catheters or instrumentation such as a tenaculum and stylet [13]. The employment of additional instrumentation has been reported to impair clinical outcomes [14]. The employment of a tenaculum facilitates the stabilization of the cervix and uterus during the entry of the catheter in the uterus, while a stylet is employed in case of a difficult and stiff cervix to guide the catheter’s entry [2,15]. These instruments require mild manipulation in order to avoid endometrial damage, the presence of pain and induction of uterine contractions due to prostaglandins releasement [16]. The incidence of embryo retention or embryo return is another aspect to be considered. The presence of mucus or blood on the transfer catheter and the technical difficulties at the time of ET have been associated with the incidence of embryo retention in the transfer catheter [17]. Immediate reload and retransfer of retained embryos constitutes a rescue method: however, there is lack of consensus on the effect of the catheter reload on reproductive outcomes [17]. In addition, adding to the issue of the vagueness regarding the definition and characteristics of a “difficult ET”, conflicting results are reported regarding the impact of the difficulty of transfer on cycle outcome, with a number of studies indicating no effect [18], while others demonstrate a significantly lower pregnancy rate [14].

Several studies on variables during and post ET have been published, while data thus far are conflicting regarding both definitions on what constitutes a difficult ET and how these parameters may affect clinical end points, leaving practitioners confounded. Moreover, the historical nature of studies investigating ET parameters may increase the potential sources of bias and confounders, leading to low-quality evidence. It is essential to concur on the effect of embryo transfer parameters, as well as the predictive value of these variables on clinical outcome. This prospective observational study aims to investigate the impact of ET parameters, such as the presence of cervical mucus in the catheter, the presence of blood, catheter reload, the employment of a tenaculum and stylet, resistance during transfer as experienced by the practitioner and discomfort as experienced by the patient. This study attempts to elucidate the degree of effect on a singular level or combined in a model. To further clarify this, the present study assesses the possible effects of each singular ET parameter as well as the effect that all possible combinations of ET parameters may exert on the IVF outcome. These data aim to identify the high-significance predictive value characteristics that merit recording, while avoiding the time-consuming redundant recording of parameters that may be unsuitable to serve as key performance indicators post ET. 

## 2. Materials and Methods

This prospective observational study was conducted between March 2018 and September 2022 in a single IVF center. A basic infertility investigation was conducted as previously described [19]. Briefly, semen analysis, hysterosalpingography and evaluation of hormone levels during the menstrual cycle combined with ultrasound screening were performed. Inclusion and exclusion criteria for this study were as follows. Couples with genetic and endocrine abnormalities, oligoasthenoteratozoospermia, severe male infertility, azoospermia, PGT cycles, sexually transmitted diseases and current or previous cancer diagnosis were excluded. Furthermore, women over 40 years old were excluded along with couples with female factor infertility, namely, tubal infertility, endometriosis, polycystic ovarian syndrome (PCOS), pelvic infection, poor ovarian reserve, premature ovarian insufficiency or cycle irregularities, as established during the basic infertility investigation. Participants included in the study presented with normal ovulation and regular length of menstrual cycles ranging from 24 to 35 days. Further inclusion criteria for the present study were normal endometrial and uterine anatomy, as assessed by hysterosalpingography. Couples presenting with idiopathic infertility or mild male factor infertility undergoing fresh autologous IVF cycles were included. 

### 2.1. Controlled Ovarian Stimulation

A common controlled ovarian stimulation protocol was applied to all recruited patients. The standard gonadotropin-releasing hormone (GnRH) long agonist protocol was employed, as previously described [20]. Briefly, 0.1 mg of GnRH agonist on day 21 of the previous cycle was initially administrated. Starting on day 2 of the menstrual cycle, patients received a daily dose of 300 IU of rFSH injections. The gonadotropin dose was adjusted according to ultrasonographical assessment of follicular development. Ovulation induction was performed employing β-hCG administration, when one or more follicles reached a diameter ≥ 17–18 mm. Serum estrogen and progesterone levels were evaluated during the treatment and ultrasound observation of the follicles was performed. Transvaginal oocyte retrieval was performed 36 h following β-hCG administration. 

### 2.2. Embryo Culture and Assessment 

Conventional IVF was performed 38 h after β-hCG injection. Conventional IVF insemination was the treatment of choice, while ICSI was performed in cases of male infertility. Following denudation, mature metaphase II (MII) oocytes were injected, while immature metaphase I (MI) and germinal vesicle (GV) oocytes were discarded. The presence of two pronuclei (2PN) as well as the extrusion of two polar bodies 16–18 h post insemination indicated normal fertilization. Day 3 embryos were evaluated from grade 1–5 according to the Veeck system [21], and subsequently, they were categorized as good, fair, and poor quality. Embryos ranked as grade 1 and 2 were considered of good quality, embryos of grade 3 were considered fair quality and embryos evaluated as grade 4 and 5 were considered poor quality embryos. Accordingly, blastocysts were assessed based on Gardner’s alphanumeric grading system. Particularly, the blastocyst is characterized from grade 1 to grade 6 according to the degree of expansion, while inner cell mass and trophectoderm quality are defined by three grades (grades A–C) according to cohesiveness and the number of cells [22]. The graded blastocysts were subsequently categorized into three categories, namely, good, fair, and poor, employing the blastocyst quality ranking tool by Zhan et al., 2020 [23]. The quality of the embryos transferred, which is one of the covariates in this study, results from a cumulative standpoint referring to the quality of the best embryo transferred. 

### 2.3. Embryo Transfer Procedure

Luteal phase support was provided to all participants as previously described [20]. As this study was performed in a clinical setting, the day of embryo transfer was decided according to the number and quality of embryos. Particularly, a cleavage-stage embryo transfer was conducted in cases with less than three embryos on day two or those presenting poor-quality embryos. In all other cases, day 4 embryos or day 5 blastocysts were transferred. In all embryo transfers, 1–3 embryos were transferred depending on total embryo number, the decision following patient–physician consultation and Greek legislation. All ETs were performed employing the same type of soft catheter to avoid confounders.

### 2.4. Outcome Measures

Several performance parameters were investigated and associated with clinical outcomes. Specifically, resistance during transfer as experienced by the physician, discomfort as experienced by the patient and the presence of blood in the catheter were investigated as ordinal variables entailing three categories “No”, “Mild” and “Significant”. The presence of cervical mucus, catheter reload, and the employment of a tenaculum and stylet were investigated as dichotomous variables entailing two categories “Yes” or “No”. In the second analysis performed focusing on the presence of blood, catheter resistance and discomfort as experienced by the patients, “mild” and “significant” were analyzed cumulatively and regarded as “yes”; therefore, these outcome measures were investigated as dichotomous variables (yes/no). The clinical outcomes evaluated were positive β-hCG, clinical pregnancy and live birth. Positive β-hCG was evaluated in maternal serum seven days post embryo transfer. Clinical pregnancy was defined by ultrasonographic visualization of at least one gestational sac 6–7 weeks following the last menstruation according to ICMART. 

### 2.5. Statistical Analyses

A univariate logistic regression was employed to evaluate the possible effect of each of the ET procedural parameters on clinical outcomes. Clinical outcomes were regarded as dependent variables and the ET performance parameters were taken as independent variables. A multivariate logistic regression model was employed to evaluate the possibility that interactions between the ET procedural parameters could affect cycle outcome. The Bonferroni correction was employed to adjust for possible multiple comparison bias regarding ET performance parameters. To assess the difference between patients with no adverse reported parameters and patients with at least one adverse parameter, the chi-square test was employed. To analyze the effect of maternal age and embryo quality on the clinical outcomes, the Cochran–Mantel–Haenszel (CMH) test was employed. The statistical analysis was performed employing R programming language. A *p*-value < 0.05 was considered statistically significant.

## 3. Results

**Figure 1:**
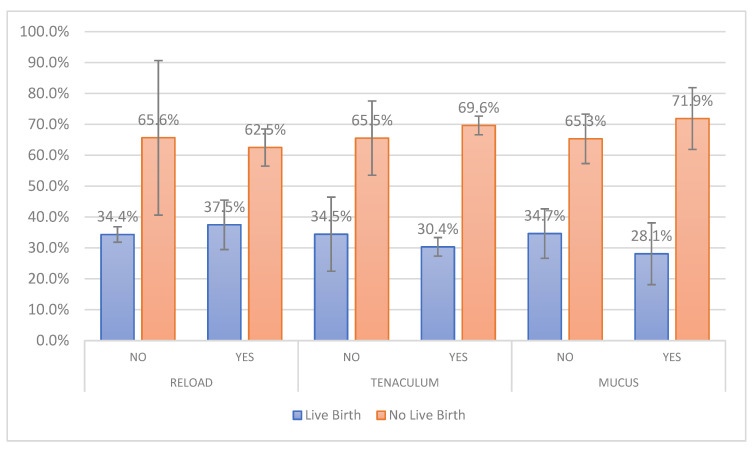
Live-birth rates according to catheter reload, use of tenaculum and observation of mucus during the ET. Error bars represent the 95% confidence intervals.

**Figure 2:**
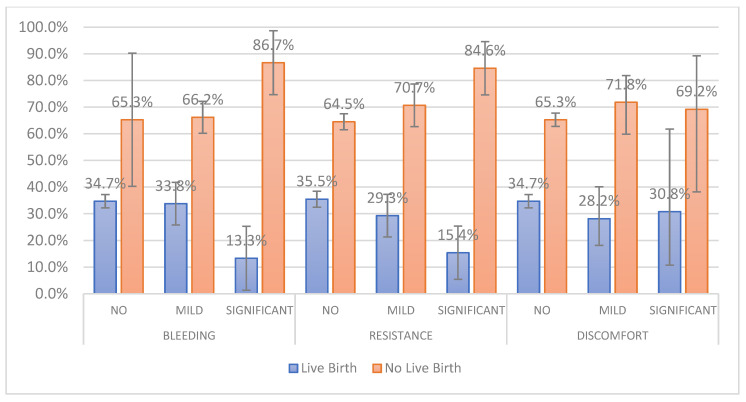
Live-birth rates according to patient’s bleeding, catheter resistance and discomfort as experienced by the patient. Error bars represent the 95% confidence intervals of the percentages.

A total of 1417 fresh autologous ETs corresponding to 1417 women who underwent IVF/ICSI cycles were recruited in this prospective observational study. The mean age of participants was 35.5 (±3.51), ranging from 20 to 39. Ultrasound guidance was employed in 1176 (82.99%) embryo transfers. Day 2 to day 6 embryo transfers were assessed, while the majority of them were performed on day 5 (53.42%). A result of a positive β-hCG was recorded in 684 women from a total of 1417 participants, while 540 women achieved a clinical pregnancy. Participants with live births were 478 (33.73%), while 51 of them (10.47%) presented with twin gestations. Patient characteristics, embryo transfer procedural parameters and clinical outcomes are presented in Table 1. The embryo transfer procedural parameters according to live-birth rates are graphically presented in Figure 1 and Figure 2.

A univariate logistic regression was performed to evaluate the possible effect of each of the ET parameters on clinical outcomes. None of the ET parameters presented with statistical significance, indicating that single ET parameters may not predict cycle outcome on a singular level. The employment of ultrasound, the number and the quality of transferred embryos, the day of transfer, maternal age and the physician performing the transfer were evaluated as covariates in a univariate regression model. The number (*p* = 0.01) and quality of transferred embryos (*p* = 0.003), as well as maternal age (*p* = 0.004), presented with statistical significance for positive β-hCG, clinical pregnancy and live-birth rate, indicating that these parameters may predict IVF cycle outcome. The results of the logistic regression for the live-birth outcome are presented in Table 2. The results of the logistic regression for the β-hCG and clinical pregnancy outcomes are presented in Supplementary Tables S1 and S2, respectively.

The multivariate approach similarly provided no association of statistical significance between ET parameters and the clinical outcomes. The results of the multivariate analysis are presented in Table 3. To evaluate the possibility that interactions between the ET parameters could affect cycle outcome, a model was created evaluating all possible interactions and combinations of the ET parameters. To adjust for possible multiple comparison bias, the Bonferroni correction was employed. Only the presence of mucus along with significant resistance seemed to present with statistical significance (*p* < 0.00001). However, when adjusting for the covariates, the combination of cervical mucus and significant resistance did not provide a statistically significant effect (*p* = 0.11) on clinical outcomes and thus cannot be considered as an independent predictor. It should be mentioned that significant resistance marginally did not reach the statistical significance threshold on all of the aforementioned occasions. 

To further the analysis, a stratification based on the embryo quality was performed. When evaluating all patients, age and live birth did not present as independent variables at each stratum of embryo quality (M^2^ = 58.84; *p* = 0.0005). On the other hand, when evaluating strictly women under the age of 35 years old, maternal age and live-birth outcome presented as independent variables at each stratum (M^2^ = 6.25; *p* = 0.27). When subgrouping only for women below the age of 35 with at least one good-quality embryo, no statistically significant association was observed for any of the outcomes evaluated (Table 4). 

A further evaluation in which the presence of blood, catheter resistance and discomfort as experienced by the patients were regarded as dichotomous variables was performed. Similar results were observed both in the univariate and in the multivariate analysis. Similarly, the presence of mucus and catheter resistance were associated with all clinical outcomes (*p* < 0.00001); however, this association was not regarded as significant following the adjustment for covariates (*p* = 0.08). When subgrouping only for women below the age of 35 with at least one good-quality embryo, no statistically significant association was observed for any of the outcomes evaluated. When evaluating the scenario in which patients did not present with any adverse parameters during ET, no statistically significant difference was observed regarding positive β-hCG rate (497/984 vs. 200/433, *p* = 0.13), clinical pregnancy rate (387/984 vs. 167/433, *p* = 0.72) or live-birth rate (351/984 vs. 136/984, *p* = 0.11) between patients with no adverse reported parameters and patients with at least one adverse parameter, respectively.

## 4. Discussion

Despite the rapid developments and the advanced technologies that have been introduced into medically assisted reproduction, implantation rates remain low. These low success rates have been attributed to several factors from embryo quality to the ET procedure. The procedure of delivering the embryos into the uterine cavity constitutes a crucial step in medically assisted reproduction, dictating implantation and clinical outcomes. Numerous studies have investigated various ET performance parameters and factors affecting IVF success rates and the results have been conflicting. The current study suggests that ET performance parameters do not seem to have a predictive value on clinical outcomes. Nevertheless, a statistical significance was observed for the presence of mucus on the catheter combined with resistance during embryo transfer. However, this combination cannot be considered an independent predictor because a statistically significant effect on clinical outcomes was not observed when adjusting for the covariates. 

The management of patients with excess cervical mucus during ET is challenging. The presence of cervical mucus increases the possibility of embryos being retained in the ET catheter [24]. Embryo retention following the initial transfer attempt is not a common event; however, it may create anxiety for both patients and practitioners as this event may jeopardize the deposition of embryos in the uterine cavity, preventing the implantation process [8,17]. Hitherto, several studies have confirmed that the presence of mucus on the catheter or the retransfer of retained embryos in the uterus does not affect the reproductive outcome, and this is based on fair evidence [8]. Our results on this matter are in concordance with published data supporting that mucus presence does not affect clinical outcomes. 

Catheter resistance was another parameter studied herein that when accompanied with mucus presence, an effect was observed, albeit muted when adjusting for covariates. Regarding catheter resistance during transfer, our data suggest that mild or significant resistance to the advancement of the ET catheter was observed in some cases (9.39% and 2.75%, respectively). However, no association was reported between resistance during transfer or discomfort as experienced by the patient and clinical outcomes. According to a recent study employing transvaginal ultrasound guidance of the catheter, it seems that there is no association between difficult transfer and pregnancy rate [25]. In contrast to our findings, several studies indicate that the degree of difficulty during embryo transfer correlates to the pregnancy rates [12]. Particularly, it has been reported that an easy transfer leads to improved clinical pregnancy rate compared to a difficult embryo transfer [12,14]. The difficulty of ET has been associated with anatomical or physiological characteristics [10]. Abnormal crypts in the cervical canal and tortuosity of the cervical canal have been suggested as the most common anatomical characteristics correlated with difficult ET, while internal contractions and pronounced anteversion of the uterus constitute less frequent causes [26]. On the matter of discomfort as experienced by the patient, our data fail to show any association with clinical endpoints in contrast to other published data. Particularly, it has been reported that pain as experienced by the patient during ET may impair clinical outcomes; in particular, reduced pregnancy rates have been observed in patients that felt pain compared to those that experienced no pain during ET [27]. Furthermore, higher pain scores were observed in non-pregnant women compared to pregnant women [27]. 

Regarding the employment of a tenaculum during ET, there have been older entries supporting a negative impact of use [28]. According to a previous study, a difficult ET requiring the employment of a tenaculum may impair clinical outcomes [29]. More recently, it has been reported that the employment of a tenaculum during ET may induce a reduction of 46% in clinical pregnancy rates [14]. However, it has been suggested that when tenaculum use is combined with an anesthetic, it does not impair ongoing pregnancy outcomes in ART cycles [30]. Nonetheless, it should be mentioned that a difficult embryo transfer followed by difficult manipulation of the catheter inside the uterus and the employment of a tenaculum or stylet may induce bleeding and the stimulation of prostaglandins and oxytocin as a response to endocervical trauma, leading to uterine contractions [10]. These contractions may jeopardize embryo deposition in the uterus [10]. The findings of the multivariate regression analysis performed in our study suggest that employment of a tenaculum cannot be an independent predictor of the cycle outcome as it does not affect negatively outcome measures. This is in discordance with published data; however, further well-designed RCTs are necessary to buttress these findings. 

Regarding the parameter of blood presence in the catheter, numerous studies indicate that the presence of blood on the transfer catheter may exert a deleterious effect on IVF cycle outcome [9]. Nevertheless, according to a systematic review and meta-analysis, a bloody catheter during embryo transfer does not seem to impair clinical pregnancy rate; however, these data have been based on low-quality evidence [12]. In concordance with these meta-analysis data, our study supports the fact that blood presence on the catheter cannot serve as an independent predictor of cycle outcome as it does not appear to detrimentally impact clinical outcomes. 

What became clear to the authors while analyzing these data and reviewing the literature is the widely employed term “difficult ET”, as described above. A point that is worth making is the fact that there is considerable discrepancy between the definitions on what constitutes a “difficult ET”. Although it appears that resistance of catheter advancement may be a common denominator, this definition is not synonymous with the term, which may lead to misconceptions and discrepancies in the scientific community. Additionally, the employment of instrumentation during ET has been involved in the definition of a “difficult ET” in numerous studies. The discomfort experienced by the patient during ET has also been reported as a component of a difficulty ET [16]. Nevertheless, it has been suggested that the parameter of discomfort may not reflect the difficulty of ET [27]. According to a study investigating the impact of discomfort during ET, no statistically significant difference for this parameter has been reported between easy and difficult ETs [25]. These contradictory findings suggest that it may not be enough to acknowledge that the term “difficult ET” may hold different meaning with regards to different characteristics and procedures that take place during ET. The heterogeneity in the definition of this term may compromise the comparison of individual studies, leading to a lack of consensus regarding the optimal ET practice. This acknowledgement led the authors of this study to investigate well-defined ET performance parameters and avoid use of the term “difficult or easy ET” in order to avoid misleading interpretations of the outcomes. 

This study highlights the essential role of maternal age, as well as the quality of transferred embryos in pregnancy outcome. Female fertility seems to decrease gradually from the age of 32 years and declines more rapidly after the age of 37 years, while the success rate of IVF seems to decrease significantly in women over 35 years old [31]. Advanced maternal age leads to fertility decline due to the decreased ovarian reserve and the reduction in oocyte quality [32]. Oocyte mitochondrial disfunction, age-related telomere shortening, cohesin dysfunctions and meiotic spindle instability have been proposed as putative mechanisms affecting oocyte competence, leading to reduced embryo developmental dynamics and increased embryo aneuploidy rate [32]. Maternal age and embryo quality are indisputably intertwined, and hence, the quality of the transferred embryos has been proposed to significantly affect implantation potential and perinatal outcomes [33,34]. It has been reported that poor-quality embryos transferred lead to lower implantation rates compared to good-quality embryos, while the day of poor-quality embryo transfer does not significantly affect the clinical outcomes [34]. Furthermore, poor-quality embryo transfer has been associated with increased miscarriage rates and lower ongoing pregnancies [35], as well as with increased risk of preterm birth and low birthweight [33]. This evidence comes in line with the findings of this study, strengthening the dogma that embryo quality and maternal age are the dominant cycle outcome predictors [36], while other parameters may serve as complimentary factors.

According to the results of our study, maternal age and embryo quality are the most predictive parameters of the IVF outcome. To compare the two and highlight their importance, an analysis of maternal age, stratified by embryo quality, has been performed. It may be observed that maternal age alone is important in patients over the age of 35, while in younger patients, embryo quality may present as a predictive parameter of higher value. This finding is in agreement with the literature. It has been reported that “average quality” blastocysts originating from women below the age of 35 present with higher live-birth rates compared to “good-quality” blastocysts originating from AMA women [37]. On the other hand, it has been established that in younger patients, embryo quality plays a more important role than maternal age [38]. Further to this, previous studies have indicated that even the age difference of a single calendar year for women over the age of 35 may significantly impact the clinical outcomes [20].

The results of this study indicate that the number of transferred embryos may affect IVF cycle outcome, as including more embryos during ET increases clinical pregnancy and live-birth rate. According to the results of our study, single embryo transfers presented with statistically significant lower positive hCG, clinical pregnancy and live-birth rates when compared to double ETs. Nonetheless, no statistically significant difference was observed between triple and double ETs. The multiple embryo transfer strategy has been adopted from clinicians to enhance pregnancy rates. According to ASRM practice guidelines, single embryo transfer constitutes the appropriate strategy to stimulate singleton gestation and decrease the number of multiple pregnancies; however, it has been recommended that poor prognosis patients may have an additional embryo transferred according to their individual circumstances [39]. Nevertheless, the literature presents conflicting results regarding pregnancy rates following multiple embryo transfers. Several studies suggest no significant differences in pregnancy and live-birth rates between single and double embryo transfer [40], while some studies report that double embryo transfer may enhance pregnancy rate and live-birth rate compared to single embryo transfer [41]. Additionally, double embryo transfer of a poor-quality embryo along with a good-quality embryo has been proposed as an alternative in low prognosis patients who may have a limited number of good-quality embryos available for transfer. It has been reported that this approach does not negatively affect clinical outcome [42]. Despite the recommendation and the scientific community adopting the elective single embryo transfer strategy (eSET), recent data support that a double ET leads to higher live-birth rates in comparison to a single ET [43]. Nonetheless, possible risks should be taken into account including multiple pregnancies and respective complications. It has been well established in the literature that twin pregnancies result in significantly adverse obstetric and perinatal outcomes and, more specifically, membrane rupture, pregnancy-related hypertension, gestational diabetes, lower birthweight and preterm birth [44]. Further to this, twin gestations have been associated with a number of congenital malformations, including chromosomal defects and circulatory and urogenital malformations [45]. Thus, it may be of upmost importance to balance the risks and benefits of single versus multiple embryo transfers. While multiple ETs may result in higher probability for pregnancy per ET and less time to achieve pregnancy, it is crucial to consider the possible complications prior to decision making on the optimal practice. Until data are collected to showcase the superiority of double ET regarding both success rates and safety, eSET should remain the optimal clinical practice. Nonetheless, in the era of precision medicine, perhaps future studies should focus on providing distinctive criteria for patient profiling on the decision-making process of the number of embryos. 

Embryo transfers following IVF can take place any day from day 2 to day 7 [3]. Numerous studies have investigated the effect of the day of embryo transfer on cycle outcome, leading to inconclusive results. According to a Cochrane review, it seems that fresh embryo transfer on day 5 compared with day 3 following oocyte retrieval may enhance live-birth and clinical pregnancy rates; however, due to low-quality evidence for live birth and moderate quality evidence for clinical pregnancy, no robust conclusions could be obtained [4]. Furthermore, studies investigating the day of embryo transfer have reported that day 5 compared with day 6 blastocyst transfers may result in higher clinical pregnancy and live-birth rates in both fresh and frozen transfers [46]. Contrary to these findings, our study indicates that the day of embryo transfer fails to affect clinical outcomes, indicating that this parameter is an invalid predictor for the IVF cycle prognosis as per data drawn herein. This is supported by a systematic review and meta-analysis of randomized controlled trials (RCTs) reporting no superiority of day 5–6 blastocyst transfer compared with day 2–3 cleavage-stage embryo transfer in clinical practice [5]. Additionally, day 4 embryo transfer has been presented as a valid option in ET decision making as no statistically significant difference has been reported between day 4 and day 2, day 3 or day 5 ET regarding clinical pregnancy rates and live-birth rates [47]. 

The multivariate regression analysis conducted herein constitutes a strong tool in controlling for confounders, providing robust results compared with other statistical tools. Nevertheless, the findings of this study should be interpreted with a degree of caution due to certain limitations. One of the primary limitations is the sample size. Although this study included a substantial number of women undergoing ET, it may still require a larger cohort presenting with each combination of parameters to reach the threshold of statistical significance. This is particularly important given the wide range of ET parameters that were assessed. Another limitation is the single-center nature of the study. While this allowed for a consistent approach to ET, it may limit the generalization and wider implications of the findings. Therefore, multicenter studies may be needed to confirm findings and ensure validity across different settings. The multiple ETs included herein constitute an additional limitation as this strategy may increase the pregnancy potential, preventing the identification of the true relationship between ET parameters and clinical outcomes.

Observation of the assessed ET procedural parameters in this study did not seem to indicate that they have a predictive value on clinical outcomes. The latest research has showcased the well-documented burnout experienced by embryologists during clinical routine practice [48]. Taking into account the time-consuming nature of recording numerous parameters in an IVF laboratory setup, and considering the burdened clinical routine practice of embryologists and how this affects burnout, this study further supports that we should only record parameters that affect clinical outcome and can serve as key parameter indicators on an evidence-based basis. If validated, these findings could have significant implications for future prediction model development. By excluding the ET parameters that do not hold predictive value, it would be possible to create a streamlined and efficient prediction model. This model would require less computational power and less dedicated time, making it more practical for use in the clinical setting.

## 5. Conclusions

Our data suggest that recording and analyzing the assessed parameters may be redundant without offering additional value in predicting the cycle’s outcome. While further studies are required to cement this finding, it appears that factors such as embryo quality and number, as well as maternal age, are the sole robust predictive factors of an IVF cycle outcome. Ultimately, ascertaining the strict consideration of parameters that can serve as true predictors and minimizing “background noise” could potentially improve the accuracy of IVF outcome predictions, thereby aiding in clinical decision making and patient counseling.

## Figures and Tables

**Table 1:** Patient characteristics (presented as mean ± standard deviation), embryo transfer parameters (presented as percentages and raw numbers) and clinical outcomes (presented as percentages and raw numbers).

ET Parameters	Mean
**Age**	35.5 (±3.51)
**Mean number of embryos transferred**	2.15 (±0.58)
**Single ETs**	10.45% (148/1417)
**Double ETs**	63.37% (898/1417)
**Triple ETs**	26.18% (371/1417)
**Quality of embryos**	
**GOOD**	22.23% (315/1417)
**FAIR**	59.50% (843/1417)
**POOR**	18.27% (259/1417)
**Day of transfer**	
**DAY 6**	6.07% (86/1417)
**DAY 5**	53.42% (757/1417)
**DAY 4**	17.3% (245/1417)
**DAY 3**	21.59% (306/1417)
**DAY 2**	1.62% (23/1417)
**Ultrasound employment**	82.99% (1176/1417)
**Resistance**	
**NO**	87.86% (1245/1417)
**MILD**	9.39% (133/1417)
**SIGNIFICANT**	2.75% (39/1417)
**Discomfort**	
**NO**	94.07% (1333/1417)
**MILD**	5.01% (71/1417)
**SIGNIFICANT**	0.92% (13/1417)
**Use of Tenaculum**	3.95% (56/1415)
**Bleeding**	
**NO**	83.95% (1189/1417)
**MILD**	15% (213/1417)
**SIGNIFICANT**	1.05% (15/1417)
**Presence of Mucus**	4.52% (64/1417)
**Catheter Reload**	0.56% (8/1417)
**Positive β-hCG**	48.27% (684/1417)
**Single ETs**	37.16% (55/148)
**Double ETs**	50.89% (457/898)
**Triple ETs**	46.36% (172/371)
**Clinical pregnancy**	38.11% (540/1417)
**Single ETs**	26.35% (39/148)
**Double ETs**	40.65% (365/898)
**Triple ETs**	36.66% (136/371)
**Live birth**	33.73% (478/1417)
**Single ETs**	26.35% (39/148)
**Double ETs**	35.97% (323/898)
**Triple ETs**	31.27% (116/371)
**Twins**	10.47% (51/478)
**Single ETs**	0% (0/39)
**Double ETs**	11.77% (38/323)
**Triple ETs**	11.21% (13/116)

**Table 2:** Results of the logistic regression.

	Estimate	Std. Error	*z* Value	*p*-Value
Maternal Age	−0.5832	0.2356	−2.721	**0.0045**
Day of ET	0.5986	0.2786	1.918	0.0613
Physician	−0.6894	1.1050	−0.624	0.5327
Embryologist	0.7056	0.4598	1.535	0.1249
Mild Bleeding	−0.1498	0.2355	−0.636	0.5747
Significant Bleeding	−1.8464	1.4317	−1.290	0.1972
Mild Resistance	0.2771	0.3272	0.847	0.3970
Significant Resistance	−0.3573	0.8058	−0.443	0.6574
Catheter Reload	1.2020	0.8697	1.382	0.1667
Employment of Tenaculum	0.4693	0.4177	1.124	0.2611
Mild Discomfort	−1.0431	1.1427	−0.913	0.3613
Significant Discomfort	−1.6868	0.9283	−1.817	0.0692
Presence of Mucus	0.2094	0.4696	0.446	0.6557
Poor-Quality Embryo	−0.6484	0.2377	−2.928	**0.0006**
Good-Quality Embryo	−0.5918	0.2366	−2.803	**0.0031**
Number of Embryos	−0.1512	0.1141	−1.325	0.1850

Values in bold indicate statistically significant difference.

**Table 3:** Results of the multivariate analysis.

	Summary Square	Mean Square	F Value	*p*-Value
Day of ET	0.673	0.67272	4.4379	**0.035340**
Maternal Age	1.612	1.61206	10.6349	**0.001139**
Embryologist	4.256	0.15764	1.04	0.408585
Physician	5.866	0.21725	1.4332	0.070346
Bleeding	0.108	0.05393	0.3558	0.700684
Resistance	0.070	0.03478	0.2295	0.794987
Reload	0.417	0.41715	2.7519	0.097378
Tenaculum	0.175	0.17467	1.1523	0.283264
Ease	0.097	0.04831	0.3187	0.727146
Discomfort	0.393	0.19641	1.2958	0.274045
Mucus	0.074	0.07351	0.4849	0.486317
Ultrasound	0.222	0.22155	1.4616	0.226894
Embryo Quality	2.017	0.67222	4.4347	**0.004142**
Number of Embryos	1.303	1.30321	3.9989	**0.015765**

Values in bold indicate statistically significant difference.

**Table 4:** Results of the logistic regression for the live-birth outcome of the subgroup of women under 35 years old and with at least one good-quality embryo.

	Estimate	Std. Error	*z* Value	*p*-Value
Maternal Age	0.07019	0.04242	1.655	0.098
Day of ET	0.08127	0.10687	0.76	0.447
Physician	−0.6894	1.1050	−0.624	0.5327
Embryologist	0.7056	0.4598	1.535	0.1249
Mild Bleeding	−0.06083	0.26036	−0.234	0.815
Significant Bleeding	−16.0603	1982.885	−0.008	0.994
Mild Resistance	0.35054	0.38269	0.916	0.36
Significant Resistance	−0.14422	0.7284	−0.198	0.843
Catheter Reload	0.05018	1.05554	0.048	0.962
Employment of Tenaculum	0.45905	0.53199	0.863	0.388
Mild Discomfort	−0.19509	0.49121	−0.397	0.691
Significant Discomfort	−16.6224	2426.842	−0.007	0.995
Presence of Mucus	0.2094	0.4696	0.446	0.6557
Top-Quality Embryo	0.25237	0.22359	1.947	0.0584
Number of Embryos	0.17532	0.18942	0.926	0.355

## Data Availability

The data underlying this article will be shared on reasonable request to the corresponding author.

## Supplementary Materials

The following supporting information can be downloaded at: https://www.mdpi.com/article/10.3390/jcm13051312/s1. Table S1: Resulkts of the logistic regression on the positive hCG outcome. Table S2: Resulkts of the logistic regression on the clinical pregnancy outcome.
