# Supplementary material for: Embryo Transfer Procedural Parameters Do Not Predict IVF Cycle Outcome
Source: J Clin Med. 2024 Feb 26;13(5):1312. doi: 10.3390/jcm13051312 (PMC10931750; doi:10.3390/jcm13051312)
Supplement: Supplementary file 1 [file jcm-13-01312-s001.zip › jcm-2769192-supplementary.pdf]

Supplementary Table S1: Resulkts of the logistic regression on the positive hCG outcome

	Estimate	Std. Error	z value	P-Value
Maternal Age	-0.6232	0.2568	-2.935	**0.0032**
Day of ET	4.2762	4.7258	0.896	0.3712
Physician	-1.4538	1.1050	-0.548	0.6978
Embryologist	0.7914	0.6982	0.994	0.3298
Mild Bleeding	-0.0359	0.1900	-0.1890	0.8500
Significant Bleeding	0.5728	0.6451	0.8880	0.3752
Mild Resistance	-0.1203	0.2478	-0.4860	0.6277
Significant Resistance	-0.6758	0.5115	-1.3210	0.1863
Catheter Reload	-0.4233	0.7910	-0.5350	0.5934
Employment of Tenaculum	-0.3417	0.3265	-1.0460	0.2956
Mild Discomfort	0.3988	0.7218	0.5520	0.5811
Significant Discomfort	0.0213	0.3371	0.0630	0.9504
Presence of Mucus	-0.3632	0.3130	-1.1600	0.2469
Poor Quality Embryo	-0.7170	0.1655	-4.3330	**<0.0001**
Good Quality Embryo	-0.2570	0.1795	-1.4320	0.1522
Number of Embryos	0.1088	0.0794	1.3700	0.1713

Supplementary Table S2: Resulkts of the logistic regression on the clinical pregnancy outcome

	Estimate	Std. Error	z value	P-Value
Maternal Age	-0.6132	0.2412	-2.879	**0.0037**
Day of ET	1.5510	0.5889	2.634	**0.0084**
Physician	-1.3985	1.3995	-0.378	0.7523
Embryologist	0.7345	0.5863	1.187	0.2731
Mild Bleeding	-0.0971	0.1960	-0.4960	0.6202
Significant Bleeding	-1.0466	0.9056	-1.1560	0.2479
Mild Resistance	0.5285	0.2644	1.9990	0.0456
Significant Resistance	-0.6111	0.5726	-1.0670	0.2859
Catheter Reload	0.1264	0.8280	0.1530	0.8787
Employment of Tenaculum	0.2978	0.3497	0.8520	0.3944

Mild Discomfort	0.2375	0.7795	0.3050	0.7606
Significant Discomfort	-0.0629	0.3650	-0.1720	0.8631
Presence of Mucus	-0.3746	0.3230	-1.1600	0.2461
Poor Quality Embryo	-0.8932	0.1806	-4.9450	**<0.0001**
Good Quality Embryo	0.2021	0.1914	1.0560	0.2908
Number of Embryos	0.0619	0.0843	0.7330	0.4633
